# Differences in Physiological Responses During Rowing and Cycle Ergometry in Elite Male Rowers

**DOI:** 10.3389/fphys.2018.01010

**Published:** 2018-07-30

**Authors:** Joshua R. Lindenthaler, Anthony J. Rice, Nathan G. Versey, Andrew J. McKune, Marijke Welvaert

**Affiliations:** ^1^Research Institute for Sport and Exercise, University of Canberra, Canberra, ACT, Australia; ^2^Sport Science, Rowing Australia, Canberra, ACT, Australia; ^3^Faculty of Health, University of Canberra, Canberra, ACT, Australia; ^4^Australian Institute of Sport, Canberra, ACT, Australia

**Keywords:** rowing, cycling, ergometry, efficiency, oxygen consumption, training prescription

## Abstract

Cycle training is an important training modality of elite rowers. Cycling is the preferred alternative to on-water and ergometer rowing as it provides a reduction in compressive forces on the thoracic cage and upper extremities while still creating a local and central acclimation to endurance training. It is hypothesised, however, that there will be differences in physiological characteristics between Concept II (CII) rowing and WattBike (WB) cycling due to the principle regarding the specificity of training that elite rowers undertake. Understanding these differences will ensure more accurate training prescription when cycling. Twenty international level male rowers, [$\dot{\text{V}}$O_2PEAK_ 5.85 ± 0.58 L.min^−1^ (CI ± 0.26 L.min^−1^)] participated in two identical discontinuous incremental exercise tests on a CII rowing and WB cycle ergometer. Ergometer modalities were randomised and counterbalanced among the group and tests occurred 7 days apart. $\dot{\text{V}}$O_2_, $\dot{\text{V}}$CO_2_, $\dot{\text{V}}$_E(STPD)_ and HR were significantly higher for every submaximal power output on the CII compared with the WB. Maximal power output on the WB was higher than on the CII [42 ± 33 W (CI ± 14 W) *p* < 0.000] but $\dot{\text{V}}$O_2PEAK_ was similar between modalities. Minute ventilation at maximal exercise was 11 L.min^−1^ lower on CII than on WB. When data were expressed relative to modality specific $\dot{\text{V}}$O_2PEAK_, power output was consistently lower on the CII as was submaximal $\dot{\text{V}}$CO_2_, RER, RPE, mechanical efficiency and BLa concentration at 75% $\dot{\text{V}}$O_2PEAK_. Across all power outputs and exercise modalities, 77% of the variance in RPE could be explained by the variance in BLa. These results demonstrate that elite rowers can attain similar $\dot{\text{V}}$O_2PEAK_ scores regardless of modality. Substantial physiological and metabolic differences are evident between CII rowing and WB cycling when power output is the independent variable with the latter being over 40 W higher. The difference in displayed power output between the ergometer modalities is attributed to differences in mechanical efficiency and a degree of power output not being accounted for on the CII ergometer. Given the lack of consistency between CII and WB power output, other physiological measures, such as HR, are better suited to prescribe WB ergometer sessions.

## Introduction

Rowing is an event held over 2000 m with races lasting 5:30 min through to 8:00 min depending on boat category. It is a predominantly aerobic endurance event where athletes with high muscle mass relative to total mass and large maximal aerobic power are successful on the international stage (Hagerman, 1984). Despite the relatively short duration of the event, rowers undertake a disproportionally large amount of training per week across various training modalities (Tran et al., 2015). In Australia, elite rowers train 17–22 h per week depending on the training phase which results in moderate improvements in maximal oxygen consumption throughout the season (Tran et al., 2015). Elite rowers utilise substantial volumes of non-specific training modalities (range: 33–48%, Tran et al., 2015) with cycling (both indoor and outdoor) forming a large component. The nature of cycling to have a low eccentric muscular load and maintain the central cardiovascular acclimation of endurance training, while substantially unloading the thoracic cage and upper extremities, normally associated with the bulk of chronic injuries in rowing (Drew et al., 2016) ensures its place as the primary non-rowing training modality.

Previous studies in rowers have shown that similar $\dot{\text{V}}$O_2PEAK_ can be attained by the same individual performing maximal exercise on a rowing and cycle ergometer (Bouckaert et al., 1983; Smith et al., 1994) despite a higher O_2_ cost of submaximal rowing (Bouckaert et al., 1983). Whether this relationship exists in elite rowers with a long training history and who undertake a significant volume of cycle training is yet to be determined. Understanding the modality specific physiological responses in elite rowers is vital to optimise exercise prescription for non-rowing training and ensure that the most desirable physiological and peripheral acclimation continue even if the rower is injured or specifically undertaking large amounts of non-rowing training.

Therefore, the aim of this investigation was to compare the submaximal and maximal physiological responses in elite male rowers to identical incremental exercise on a rowing and cycle ergometer to more accurately prescribe their off-water training on the cycle ergometer.

## Materials and Methods

### Participants

Twenty-two international class male rowers agreed to participate in this investigation. Two participants were excluded at the completion of data collection for breaches of the prescribed pre-test protocols. All participants were in training at the National Training Centre in Canberra and represented Australia at the 2017 Rowing World Championships. Among the group were four current World Champions, three former World Champions and three who competed at the 2016 Rio Olympic Games with one winning a silver medal. All participants were undertaking regular rowing and cycle ergometry and were considered to be exceptionally familiar with both ergometers and modalities. The study was approved by the University of Canberra Human Ethics Committee (HREC 17-63) and each participant provided signed informed consent prior to beginning any data collection.

### Experiment Design

The study composed of two separate, seven-stage discontinuous incremental exercise protocols undertaken 1 week apart. Prior to the first session, participants were assigned into two groups and randomly allocated an ergometer modality [Concept II (CII) rowing ergometer or WattBike (WB) cycling ergometer] for the first incremental test. Participant numbers were balanced across the ergometers and ergometer modality was crossed over for the second trial 7 days later. All athletes undertook similar training in the 14-day period surrounding the study.

#### Test Procedure

All tests were undertaken at a quality assured physiological testing laboratory (Australian Institute of Sport, Belconnen ACT, Australia). Upon arrival, participant’s mass was recorded followed by a resting blood lactate (BLa) measure obtained from an earlobe (The Edge Handheld Lactate Analyser, APEX Biotechnology Corp., Taiwan). Reliability of these handheld analysers has previously been determined in this laboratory (Bonaventura et al., 2015). Prior to the start of each test, environmental conditions were measured and recorded (Vaisala PTU301, Vaisala Corporation, Finland). Participants wore a heart rate (HR) monitor (Premium HRM, Garmin Ltd., United States) to record HR. Rating of perceived exertion (RPE) was determined using a 6–20 Likert scale (Borg, 1970).

#### Measurement of $\dot{\text{V}}$O_2_

A one-way breathing valve (Model 6700, Hans Rudolph, United States) and mouthpiece were attached to the participant using a customised headset. The expiratory port of the one-way valve was connected via a 1.5-m large bore hose to a fully automated custom-built, open-circuit indirect calorimetry system with associated in-house software (Australian Institute of Sport, Belconnen ACT, Australia). All respiratory gases were collected in 120-L gas impermeable foil bags which alternated continuously every 30 s. Minute ventilation [$\dot{\text{V}}$_E(STPD)_] was measured directly by evacuation of each bag while O_2_ and CO_2_ concentrations were continuously sampled during evacuations using analysers calibrated at the start and end of each test (S-3A/I with N-22M O_2_ sensor and CD-3A with P-6-1B CO_2_ sensor, AEI Technologies Inc., United States). Immediately prior to beginning exercise, a piece of flexible tape was placed across the bridge of the participant’s nose to prevent the nose clip from slipping during the test. Technical specifications of the system have been described previously (Saunders et al., 2004). The metabolic cart began recording data 30 s prior to the athlete beginning. Respiratory variables were continuously collected during all workloads, with participants being allowed to remove the mouthpiece during the 1-min rest interval. Submaximal respiratory data were recorded as the mean value of the final two 30-s measurements. $\dot{\text{V}}$O_2PEAK_ (L.min^−1^) in the final step was identified as the largest combined value for two consecutive 30-s samples occurring anywhere in the final 4-min step.

#### Equipment

Rowing trials were conducted on a Concept II rowing ergometer (CII: Concept II model D; Concept2 Inc., United States). As the CII cannot be mechanically calibrated, the drag factor was set for each participant according to their weight category (130 for heavyweights; 120 for lightweights). Prior to the start of each rowing test, the work monitor was pre-programmed with the work:rest periods and the athlete’s HR monitor was wirelessly connected. Data were collected simultaneously and averaged on a stroke by stroke basis. Cycle ergometry tests were undertaken on a magnetically and air-braked ergometer (WB; WattBike Pro; WattBike, United Kingdom), which was mechanically calibrated using a first principles dynamic calibration rig (Australian Institute of Sport, Belconnen ACT, Australia). The WB was pre-programmed with work:rest intervals but was manually stopped and started at each increment such that data were collected for the entire 4-min period and averaged correctly. A fan was utilised for all tests and was located 1 m in front and to the left of the ergometer, it was set on 200 rpm for the first incremental step, 300 rpm for step 2 and 400 rpm for steps 3–7.

#### Incremental Exercise Protocol

An identical seven-stage discontinuous incremental protocol was used to assess fitness and performance on both ergometers (Rice and Osborne, 2013). The test protocol consisted of seven increasing workloads of 4-min duration, with 1-min rest/recovery period between each step. The first six steps of the protocol had each subject working at set workloads according to their season best CII 2000 m ergometer rowing score. Athletes were required to maintain the required power for each workload, which due to their familiarity to both ergometers and the exercise intensity being submaximal was a straight forward task. The seventh and final step of the protocol was a performance trial where each subject was asked to cover as much distance as possible in the 4 min (time trial format). For each individual rower, the incremental power outputs for the WB were identical to the CII incremental power outputs. Physiological variables were collected during each 4-min period with power output, and stroke rate/cadence averaged across the entire 4 min. HR for the submaximal steps was taken as the average of the final 30 s of each step. For the final step, HR_PEAK_ was taken as the highest recorded value for the 4-min period. RPE and BLa were measured during the 1-min period between each workload. BLa for the final maximal step was measured immediately post exercise and then taken every 4 min post, until an increase of no more than 1.0 mmol.L^−1^ in BLa was observed. Peak BLa was taken as the highest value obtained post maximal exercise and used in all analyses.

#### 24-h Food Intake and Anthropometry

For the 24-h period preceding each ergometer trial, participants were required to complete a food diary. In the days leading into the second ergometer trial, participants were reminded to follow as closely as possible the 24-h food intake recorded for the first ergometer trial. Twenty-four-hour food recordings were analysed for total energy and macronutrient intake by an accredited practising dietician using FoodWorks Professional v7.0.3016 (Xyris Software Pty Ltd., Australia). Anthropometric measures (height, mass and sum of seven skinfolds) were collected during routine monitoring at the National Training Centre and were undertaken in a 1-week window either before or after the completion of the investigation.

### Statistical Analysis

Data were represented in two distinct ways. In the first instance, WB and CII data were represented using power output (W) as the consistent independent variable for both ergometers. Increasing power outputs were analysed across all dependent variables [expired minute ventilation ($\dot{\text{V}}$_E(STPD)_), volume of O_2_ consumption ($\dot{\text{V}}$O_2_), volume of CO_2_ production ($\dot{\text{V}}$CO_2_), respiratory exchange ratio (RER), HR, BLa and RPE]. Once apparent that submaximal $\dot{\text{V}}$O_2_ was substantially different between ergometer modality at any given power output, all data were re-examined.

Given $\dot{\text{V}}$O_2PEAK_ did not differ between the two ergometer modalities, the second method used to analyse the data consisted of expressing metabolic and perception-based dependent variables on the WB and CII at three equivalent relative $\dot{\text{V}}$O_2_ intensities (50, 75 and 100% modality specific $\dot{\text{V}}$O_2PEAK_). All associated dependent variables were then calculated from the appropriate curve fit for their relationship with $\dot{\text{V}}$O_2_ across both ergometer modalities.

Gross mechanical efficiency (GME) was calculated using standard equations [energy output in kCal (power output (W) × 0.86/60) divided by energy in ($\dot{\text{V}}$O_2_ × RER kCal equivalent per LO_2_)] (Gaesser and Brooks, 1975). Data are expressed as mean ±*SD* and ±95% confidence interval (CI). A paired *T*-test was performed across all variables and statistical significance was accepted at a *p*-level of ≤0.05 [Microsoft Excel for Mac (2017)]. Original *p*-values were corrected for multiple testing by stabilising the false discovery rate using the Benjamini–Hochberg correction method (Benjamini and Hochberg, 1995). All reported *p*-values have been adjusted.

## Results

There was no significant difference in 24-h food intake prior to each incremental test (*p* = 0.416). Group anthropometric data are presented in **Table 1**. Individual peak values determined during either the WB or CII maximal step are indicative of highly trained endurance athletes undertaking maximal exercise; $\dot{\text{V}}$O_2PEAK_ (5.85 ± 0.58, CI ± 0.26 L.min^−1^; 66.3 ± 3.6, CI ± 1.6 mL.min^−1^.kg^−1^), Power_PEAK_ (479 ± 53W, CI ± 23W), HR_PEAK_ (190 ± 8, CI ± 4 b.min^−1^), BLa_PEAK_ (16.5 ± 4.2, CI ± 1.8 mmol.L^−1^), V_E(STPD)_ (161.9 ± 19.0, CI ± 8.4 L.min^−1^) and RER (1.10 ± 0.07, CI ± 0.03).

**Table 1:** Group anthropometric data obtained within 1 week of the testing period. Body mass index [BMI; weight (kg)/height^2^ (m)].

	Mean	*SD*	95% CI
Age (years)	23.8	1.6	0.7
Height (cm)	190.8	7.3	3.1
Weight (kg)	88.9	9.8	4.2
BMI	24.3	1.7	0.8
Sum of seven skinfold (mm)	48.5	9.9	4.4

### Submaximal Exercise

**FIGURE 1:**
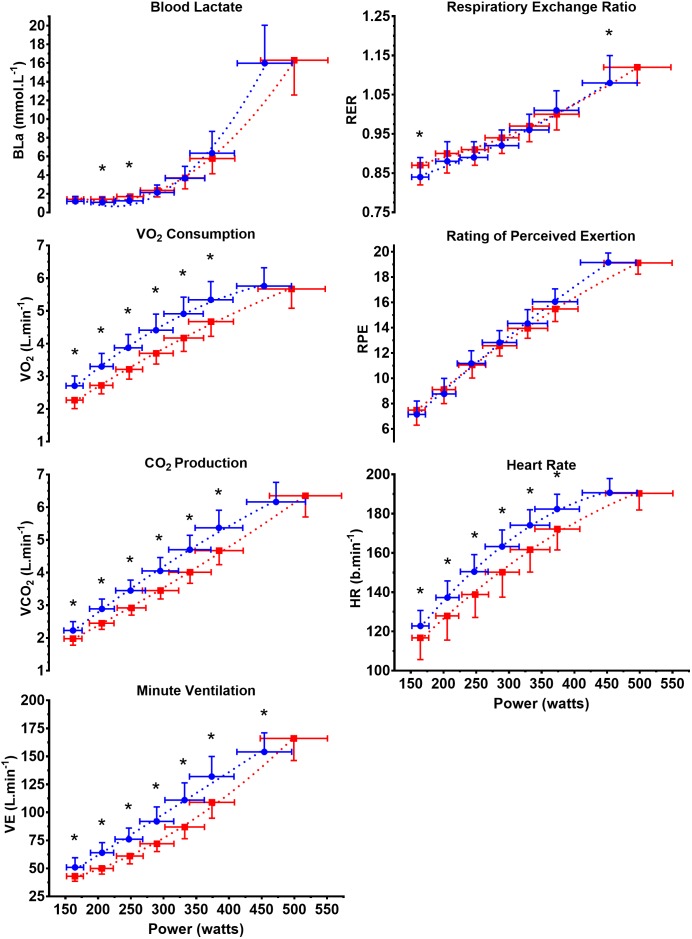
Metabolic and perception variables expressed relative to power output on the Concept II (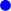) and WattBike (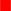). *X* and *Y* data are mean ± *SD*. BLa, blood lactate concentration; $\dot{\text{V}}$O_2_, volume of oxygen consumption; $\dot{\text{V}}$CO_2_, volume of carbon dioxide production; $\dot{\text{V}}$_E(STPD)_, expired minute ventilation; RER, respiratory exchange ratio; RPE, rating of perceived exertion; HR, heart rate. ^∗^ Indicates significance between CII and WB at discrete power outputs (incremental steps). No differences between CII and WB power output were measured at any of the seven incremental steps.

**FIGURE 2:**
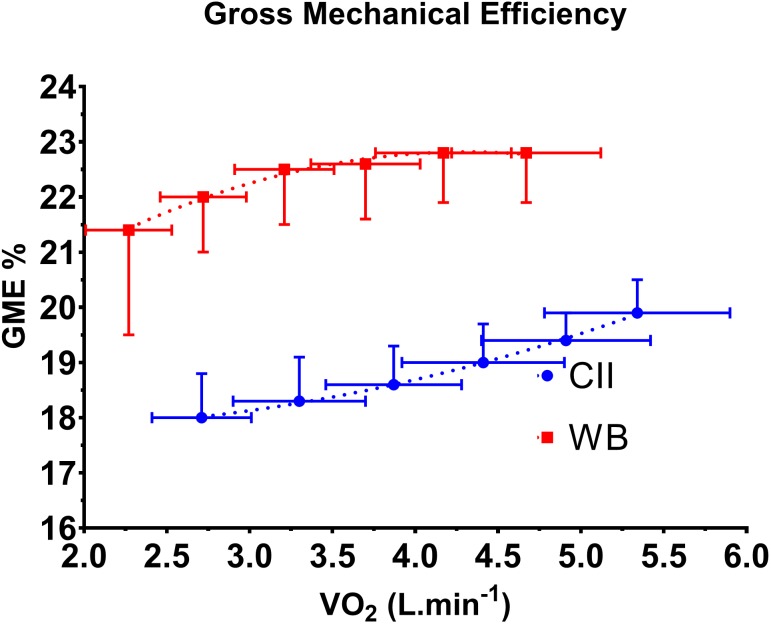
Gross mechanical efficiency (GME %) expressed relative to power output (W) on the Concept II (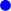) and WattBike (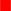). *X* and *Y* data are mean ± SD. ^∗^ Indicates significance between CII and WB at discrete power outputs (submaximal incremental steps).

**FIGURE 3:**
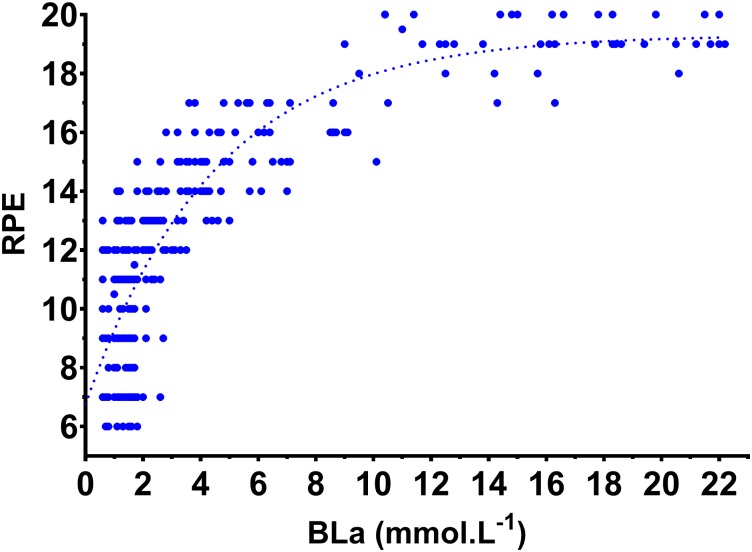
Association between blood lactate concentration (BLa) and rating of perceived exertion (RPE) for all workloads across both CII and WB ergometers. *R*^2^ = 0.771.

Power outputs during the six submaximal stages of the incremental protocol were not different between the WB and CII ergometers. **Figure 1** displays CII and WB power output against corresponding values for BLa, $\dot{\text{V}}$O_2_, $\dot{\text{V}}$CO_2_, $\dot{\text{V}}$_E(STDPD)_, RER, RPE and HR. For any given power output CII $\dot{\text{V}}$O_2_, $\dot{\text{V}}$CO_2_, $\dot{\text{V}}$_E(STPD)_ and HR were significantly higher (*p* ≤ 0.002) during submaximal exercise (incremental steps 1–6) with a significantly corresponding lower GME (range of difference 2.9–3.9%; *p* < 0.000, **Figure 2**). BLa and RPE show very similar relationships to submaximal power output on both the WB and CII ergometers with only BLa on the CII for steps 2 and 3 being significantly lower than WB (*p* = 0.029 and *p* = 0.012, respectively). Although non-significant, there was a consistent trend for the WB to display slightly lower BLa and RPE in the final submaximal step compared with the CII (5.7 vs. 6.2 mmol.L^−1^, *p* = 0.345 and 15.6 vs. 16.1 RPE, *p* = 0.054, respectively). When examined as an association to each other, there was a logarithmic relationship with 64 and 57% of the variance in RPE explained by the variance in BLa on the WB and CII ergometers, respectively. When data from all workloads across both modalities were combined, there was a very strong association between the BLa and RPE with 77% of the variance in RPE explained by BLa (**Figure 3**).

### Maximal Exercise

Maximal power output on the WB was higher than maximal power output on the CII (42 ± 32 W, (CI ± 14 W), *p* < 0.000). BLa, $\dot{\text{V}}$O_2_, $\dot{\text{V}}$CO_2_, HR and RPE (**Figure 1**) were similar between the two exercise modalities. $\dot{\text{V}}$_E(STPD)_ and RER (**Figure 1**) were significantly higher on the WB during maximal exercise when compared with CII rowing (*p* < 0.000 and *p* = 0.033, respectively).

### Variables Expressed Relative to Submaximal $\dot{\text{V}}$O_2_

**FIGURE 4:**
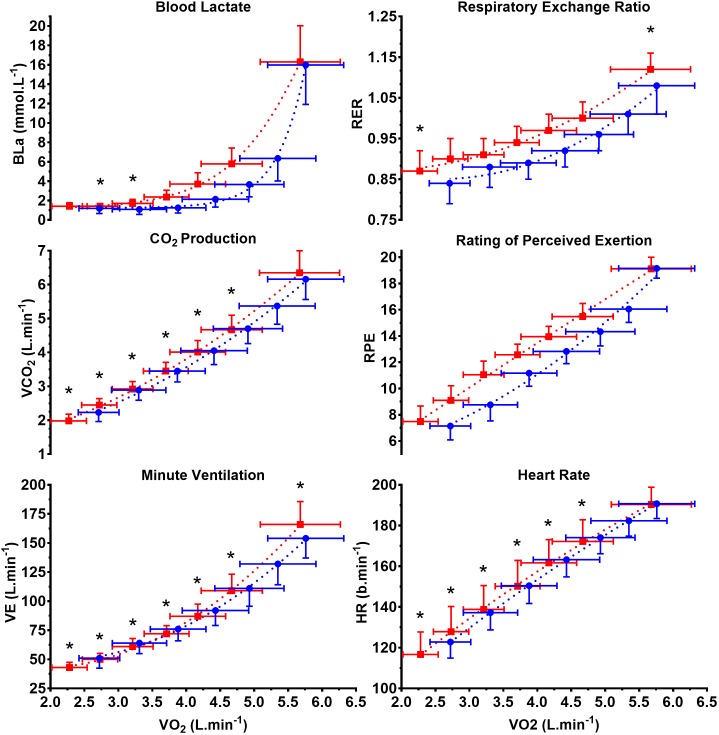
Metabolic and perception variables expressed relative to oxygen consumption on the Concept II (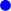) and WattBike (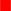). *X* and *Y* data are mean ±*SD*. BLa, blood lactate concentration; $\dot{\text{V}}$CO_2_, volume of carbon dioxide production; $\dot{\text{V}}$_E(STPD)_, expired minute ventilation; RER, respiratory exchange ratio; RPE, rating of perceived exertion; HR, heart rate. ^∗^ Indicates significance between CII and WB at discrete power outputs (incremental steps).

**Figure 4** displays BLa, $\dot{\text{V}}$CO_2_, $\dot{\text{V}}$_E(STPD)_, RER, RPE, and HR on both ergometers with respect to modality specific $\dot{\text{V}}$O_2_. During submaximal exercise, there was a substantial right shift in the BLa/$\dot{\text{V}}$O_2_ and RPE/$\dot{\text{V}}$O_2_ curve for exercise on the CII, indicating lower BLa and RPE values for most submaximal $\dot{\text{V}}$O_2_ while rowing. HR and $\dot{\text{V}}$_E(STPD)_ were very similar at the same submaximal $\dot{\text{V}}$O_2_ on the WB and CII, while there was a consistent trend for cycling $\dot{\text{V}}$CO_2_ to be higher than rowing at similar $\dot{\text{V}}$O_2_.

### Differences Between WB and CII at Equal % of Modality Specific $\dot{\text{V}}$O_2PEAK_

Mean ±*SD*, CI and *p*-value at 50, 75 and 100% $\dot{\text{V}}$O_2PEAK_ for all dependent variables on the WB and CII ergometers are shown in **Table 2**. Power output on the WB was significantly higher (*p* < 0.000) at all three comparative exercise intensities (range 40–56 W). Submaximal $\dot{\text{V}}$CO_2_ and RER were significantly lower ($\dot{\text{V}}$CO_2_: 50 and 75%; RER: 50 and 75%) on the CII compared with the WB and despite being lower during maximal exercise, this was not statistically significant compared with maximal exercise on the WB (maximal $\dot{\text{V}}$CO_2_: *p* = 0.092; RER: *p* = 0.074). HR differed by less than 3 b.min^−1^ across all modality specific $\dot{\text{V}}$O_2_ (50% *p* = 0.158, 75% *p* = 0.117 and 100% *p* = 0.584). At 100% $\dot{\text{V}}$O_2PEAK_, $\dot{\text{V}}$_E(STPD)_ was significantly lower on the CII by ∼11.0 L.min^−1^, (*p* < 0.000). At 75% $\dot{\text{V}}$O_2PEAK,_ BLa was higher on the WB than CII (*p* < 0.000) but were similar between both ergometers at 50 and 100% $\dot{\text{V}}$O_2PEAK._ WB RPE was significantly higher at 50 and 75% of $\dot{\text{V}}$O_2PEAK_ compared with CII (50% *p* < 0.000 and 75% *p* < 0.000), but was similar during maximal exercise. GME was significantly lower (range 2.4–4.1%) across submaximal intensities on the CII (50% *p* < 0.000 and 75% *p* < 0.000).

**Table 2:** Differences in metabolic variables across three discrete gross mechanical efficiency $\dot{\text{V}}$O_2_. CII, concept 2 rowing ergometer; WB, WattBike cycle ergometer; Diff., difference score; $\dot{\text{V}}$O_2_, volume of oxygen consumption; $\dot{\text{V}}$CO_2_, volume of carbon dioxide production; $\dot{\text{V}}$_E(STPD)_, expired minute ventilation; RER, respiratory exchange ratio; RPE, rating of perceived exertion; GME, gross mechanical efficiency (no 100% value as anaerobic metabolism was not calculated for).

		50% $\dot{\text{V}}$O_2PEAK_				75% $\dot{\text{V}}$O_2PEAK_				100% $\dot{\text{V}}$O_2PEAK_			
		Mean	± SD	95% CI	*p*-Value	Mean	± SD	95% CI	*p*-Value	Mean	± SD	95% CI	*p*-Value
Power output (W)	CII	175	18	8		281	23	10		454	42	18	
	WB	215	24	11		338	34	15		496	51	23	
	Diff.	−40	18	8	0.000^∗^	−57	17	7	0.000^∗^	−42	33	14	0.000^∗^
$\dot{\text{V}}$O_2_ (L.min^−1^)	CII	2.88	0.28	0.12		4.32	0.42	0.18		5.76	0.56	0.24	
	WB	2.84	0.29	0.13		4.26	0.44	0.19		5.67	0.59	0.29	
	Diff.	0.04	0.16	0.07	0.335	0.06	0.24	0.11	0.335	0.08	0.33	0.14	0.335
$\dot{\text{V}}$CO_2_ (L.min^−1^)	CII	2.43	0.24	0.10		3.92	0.31	0.14		6.17	0.60	0.26	
	WB	2.56	0.21	0.09		4.11	0.34	0.15		6.35	0.65	0.29	
	Diff.	−0.09	0.15	0.07	0.004^∗^	−0.16	0.15	0.07	0.000^∗^	−0.19	0.42	0.18	0.092
$\dot{\text{V}}$_E(STPD)_ (L.min^−1^)	CII	54.8	8.2	3.6		88.8	12.7	5.6		154.4	17.00	7.4	
	WB	52.9	5.8	2.5		90.8	10.1	4.4		165.8	19.7	8.6	
	Diff.	2.5	8.3	3.64	0.290	−1.7	10.6	4.46	0.454	−11.4	8.7	3.80	0.000^∗^
RER	CII	0.85	0.05	0.02		0.91	0.04	0.02		1.08	0.07	0.03	
	WB	0.90	0.05	0.02		0.97	0.03	0.01		1.12	0.04	0.01	
	Diff.	−0.04	0.04	0.02	0.002^∗^	−0.05	0.03	0.01	0.000^∗^	−0.04	0.09	0.04	0.074
BLa (mmol.L^−1^)	CII	1.2	0.5	0.2		1.7	0.6	0.3		16.0	4.1	1.8	
	WB	1.4	0.3	0.1		3.9	0.9	0.4		16.2	3.7	1.6	
	Diff.	−0.3	0.6	0.3	0.131	−2.2	1.1	0.5	0.000^∗^	−0.2	3.7	1.61	0.816
HR (b.min^−1^)	CII	128	11	5		161	10	5		191	7	3	
	WB	130	12	5		164	12	5		190	9	4	
	Diff.	−3	7	3	0.158	−2	6	2	0.117	0	3	1	0.584
RPE	CII	7.5	1.1	0.0		12.5	1.0	0.0		19	0.5	0.0	
	WB	9.5	1.0	0.0		14.5	1.0	0.0		19	1.0	0.0	
	Diff.	−2.0	0.8	0.4	0.000^∗^	−1.9	0.8	0.3	0.000^∗^	0.1	1.1	0.5	0.674
GME (%)	CII	18.1	1.0	0.4		18.6	0.7	0.3					
	WB	22.1	1.1	0.5		22.6	0.9	0.4					
	Diff.	−4.1	1.4	0.6	0.000^∗^	−4.0	1.0	0.4	0.000^∗^				

*^∗^Significant P < 0.05.*

## Discussion

The results presented here show that significant differences exist in many variables at submaximal power outputs, especially with respect to key metabolic variables such as $\dot{\text{V}}$O_2_, $\dot{\text{V}}$_E(STPD)_ and HR. When the data were analysed with respect to modality specific peak oxygen consumption, the CII power output and GME were significantly lower during both submaximal and maximal exercise. Submaximal RPE was higher on the WB as was BLa at 75% VO_2PEAK_. During maximal exercise, rowers ventilated significantly less on the CII ergometer despite achieving a similar $\dot{\text{V}}$O_2PEAK_ on the WB. These data demonstrate that metabolism on the WB and CII ergometer at similar displayed power output is different and must be accounted for during training prescription, if specificity of training intensity is required.

One of the most intriguing results from this study is the significantly different relationship between power and oxygen consumption for the same athlete performing exercise on the WB and CII ergometer. There was a consistently higher power (>40 W) on the WB for any given $\dot{\text{V}}$O_2_ compared with the same individual rowing. Given that the same metabolic cart was used for all testing and the cart was calibrated with three known high precision grade gases immediately before and after each test, as well as using a displacement transducer to evacuate each bag in order to directly measure minute ventilation, we are confident that the metabolic cart performed to the highest precision available. To further support our belief that the metabolic cart was not responsible for the observed differences in the power/$\dot{\text{V}}$O_2_ relationship between the ergometer modalities, we compared our current data with metabolic data from our laboratory measured on a different metabolic cart using an unknown number of different cycle and rowing ergometers and highly trained athlete populations. Despite the variation in participants and ergometer models (both rowing and cycling), the current WB and CII data are highly comparable to our previously collected data.

We have confirmed work from other authors when demonstrating that GME is different in the same individual performing rowing and cycling ergometry (Cunningham et al., 1974; Hagerman et al., 1978; Bunc and Leso, 1993). Despite both movements being cyclical in nature, rowing involves a largely different duty cycle and activated muscle mass compared with that of cycling and this is suggested to contribute to the lower efficiency while rowing (Di Prampero et al., 1971; Steinacker et al., 1986). In our study, the rowers demonstrated an increase in GME during incremental work on both ergometers. This could be due to an increase in duty cycle, especially on the CII ergometer. On the WB, rowers predominantly chose lower cadences for the first two to three workloads, and then settled on a consistent cadence for the remainder of the test which may explain the plateau in GME beyond 250 W power output on the WB (∼22.5% efficient, see **Figure 2**). In contrast, the GME while rowing increased in a linear fashion throughout the test such that there was ∼2% change in GME during the submaximal components of the protocol, peaking at 19.9% during the sixth increment (372 ± 34, CI ± 15 W). These trends are consistent with others (Di Prampero et al., 1971; Steinacker et al., 1986), with our GME measures lying in the middle of the range of other reports which have shown 14–26% rowing efficiency in rowers (Di Prampero et al., 1971; Cunningham et al., 1974; Hagerman, 1984; Steinacker et al., 1986; Bunc and Leso, 1993).

The rowing stroke is structured into two distinct phases; drive and recovery. To increase power output during an incremental test, a rower can choose to increase the force exerted on the handle during the drive phase, increase the stroke rate, or a combination of both. Our experience suggests that highly trained rowers in an unfatigued state (as in our study), typically increase power output using the combination of high handle force and a higher stroke rate, rather than use increases in handle force alone to increase power output. Because of this self-selected strategy, increases in power output typically occur via increases in stroke rate which happens through a combination of decreasing both drive time and recovery time as stroke rate/power output increases. Kleshnev (2003) reported that at a stroke rate of 16 s.min^−1^, a single sculler will have a drive time of 1.4 s and a recovery time of 2.3 s. At 30 s.min^−1^, this changes to 1.0 and 1.0 s for drive and recovery, respectively. This example can explain why GME increases in rowing throughout the range of stroke rates used during our incremental protocol. Specifically, drive time remains relatively constant, with increases in power output achieved primarily through a progressive decrease in recovery time of each stroke. Based on this, the work:rest ratio balances out, and the efficiency of that movement improves, hence the progressive increase in GME while rowing throughout an incremental protocol.

We have shown a main effect difference for GME between rowing and cycle ergometry up to 4.5%. This will account for some of the difference in $\dot{\text{V}}$O_2_ at any given submaximal power output on the CII ergometer when compared with the corresponding power on the WB. However, the lower GME cannot account for all the difference in $\dot{\text{V}}$O_2_ between the ergometers and leaves the authors to believe that there is work on the CII ergometer that is unaccounted for in the display of power output. The investigation of this hypothesis is beyond the scope of our data but given that both power output/$\dot{\text{V}}$O_2_ relationships on the WB and CII are linear and differ by a similar offset across the range of submaximal power outputs suggests that there is a systematic difference which is likely to be accounted for by differences in GME and a better representation of all work done on the CII ergometer. The authors speculate that given the method by which power output is calculated on the CII ergometer that work done shifting the rower’s body mass during both the drive and recovery phases consumes O_2_ but has little to no power output associated with it (0 W during the recovery phase). This helps explain the higher $\dot{\text{V}}$O_2_ while rowing at all submaximal powers compared with WB cycling. When expressed relative to a consistent %$\dot{\text{V}}$O_2PEAK_ there is ∼45 W power differential between the ergometer modalities and when differences in GME are accounted for the remaining difference in wattage must be assumed to be unaccounted power on the CII ergometer.

One of the original purposes of this study was to accurately measure the differences in metabolic variables and effort perception of submaximal and maximal work done on the CII and WB to more accurately prescribe training for rowers while they are on the WB. Rather than provide clear suggestions for load adjustment on the WB to equal similar work on the CII ergometer, the authors feel the current data has added a layer of confusion rather than clarity and forced them to question the true stimulus for training acclimation. Our data demonstrate that if power output is assumed to be accurately represented on both the WB and CII work monitors, then BLa and RPE are similar across all seven steps of our protocol. Conversely, HR, $\dot{\text{V}}$_E(STPD)_, $\dot{\text{V}}$O_2_ and $\dot{\text{V}}$CO_2_ values are all higher on the CII ergometer at any of the six submaximal workloads compared with the corresponding power outputs on the WB. The consistent variables that are used to prescribe and evaluate training in the daily training environment of rowers are BLa, RPE, HR and power output with the latter being the only external load indicator. Our data demonstrate that the responses of these variables to the identical displayed wattage are not consistent and more importantly, that when the data are rearranged and represented relative to oxygen consumption; a measure that the authors believe is a more accurate measure of total body work across any training modality and most likely a stronger variable to link to training acclimation than is BLa or RPE, that substantial differences in BLa and RPE are shown while HR, $\dot{\text{V}}$_E(STPD)_ and $\dot{\text{V}}$CO_2_ normalise, a similar correlation has been shown by Basset and Boulay (2000, 2003) with HR and $\dot{\text{V}}$O_2_ when expressed as a percentage of $\dot{\text{V}}$O_2MAX_ between cycle ergometer and treadmill tests. The question then remains as to what easily monitored variable should be used to prescribe routine cycle training in the daily rowing environment? Given the strong linear association between HR and $\dot{\text{V}}$O_2_ across both ergometer modalities, it is likely that HR is the best surrogate for a direct measure of $\dot{\text{V}}$O_2._ BLa and RPE in this instance are higher on the WB than CII ergometer at any given HR or $\dot{\text{V}}$O_2_. This is likely a result of less muscle mass activated to complete similar metabolic work, thus creating local muscular acidity and a higher perception of effort in cycling. Whether greater conditioning to cycling would decrease the difference in BLa and RPE between WB and CII remains to be investigated, but it is acknowledged that the elite rowers used in this investigation are significantly more conditioned for rowing than cycling, despite being able to reach similar peak values in either modality and undertaking considerable volume of work on the WB.

One of the variables that showed a stark difference during the maximal step between both ergometers was $\dot{\text{V}}$_E(STPD)_ with CII ergometry having >11 L.min^−1^ less ventilation than WB ergometry. This has been noted before by Smith et al. (1994) and shows the unique constraints that are placed on a rower’s ventilation during maximal exercise. Due to the mechanical limitations placed on the rower while attempting to take a deep breath in a crouched/cramped position, as well as the Valsalva manoeuvres that need to occur while thoracic bracing takes place during both the drive and early and late recovery phases of the stroke, ensure that minute ventilation cannot meet the O_2_ demands. As such, while rowing maximally O_2_ extraction at the tissue level compensates to ensure sufficient aerobic metabolism. In our experience, the relative under ventilation during maximal exercise on the CII primarily due to mechanical restraints placed on the thoracic cage results in a significant overshoot of expired ventilation immediately post exercise in order to stabilise blood and muscle acidity levels as rapidly as possible. Although not measured in the present study, it was noted how quickly rowers in this investigation recovered from maximal WB exercise when compared with maximal CII exercise, despite similar $\dot{\text{V}}$O_2PEAK_, RPE and BLa.

Our data showed a similar relationship between power output and RPE on both the CII and WB ergometers. When this relationship was examined relative to submaximal $\dot{\text{V}}$O_2_, the CII ergometer elicited a lower RPE for any given $\dot{\text{V}}$O_2_ and only at maximal exercise did the two ergometer modalities converge. This is an interesting point in that ratings of perceived exertion do not appear to be linked to total body work regardless of exercise modality. However, if our RPE data are represented relative to BLa concentration, the relationships become increasingly similar during either modality suggesting, similarly to Hetzler et al. (1991), that strong to very strong relationships exist between RPE and BLa, regardless of exercise modality.

The CII ergometers are very difficult to calibrate with externally driven mechanical devices. The work monitor of the CII does provide the ability to check the drag factor of the flywheel where the resistance is created but the measure is simply displaying the rate of deceleration of the flywheel after each drive phase of the stroke is completed. The drag factor check the rate of deceleration of the fan wheel according to the current ambient conditions and ensures that similar values are used between different CII ergometers or on the same CII ergometer across different days. This, however, is not a calibration of the power output value displayed on the work monitor, as that is coded internally and generic among all CII rowing ergometers. The most accepted method to accurately validate the power output displayed on the work monitor is to attach a load transducer to the last chain link between the chain and ergometer handle and have a linear displacement transducer measure the chain velocity during the drive phase of each stroke. We were unable to achieve this level of precision for our investigation and so it remains possible that our power output data on the CII are incorrect. The CII ergometer used in this investigation was near new and in excellent mechanical condition. As with the WB, it was not used during the course of the investigation for anything other than testing our cohort.

One of the major aims of this investigation was to understand the metabolic and physiological differences between rowing and cycling for international class rowers in order to better prescribe cycling training for them when they are injured or undertaking heavy indoor cycling blocks in their training. The data presented in this investigation raise significant questions around which variables are key to anchor cycling training prescription on. Our data demonstrate that power output on the cycling and rowing ergometers do not elicit the same central oxygen demand [as indicated by $\dot{\text{V}}$O_2_, HR and $\dot{\text{V}}$_E(STPD)_]. In contrast, equivalent wattages on the rowing and cycling ergometer do elicit the same RPE and BLa responses and that the latter two variables are tightly linked regardless of exercise modality. In light of the differences we measured between rowing and cycling ergometry, the question remains as to what is the best variable to anchor training prescription on; $\dot{\text{V}}$O_2_ or power output? It is the belief of the authors that given rowing performance and oxygen consumption are highly correlated (Secher et al., 1982; Hagerman and Staron, 1983; Steinacker, 1993) that this variable should be the key variable by which to prescribe training. Given the difficulty in the routine measure of this variable in the daily training environment and the strong relationship between HR and $\dot{\text{V}}$O_2_ regardless of modality in the present study, it would seem HR to be the simplest and most readily available variable by which to prescribe cycling training. Prescription using this variable, however, will come at a cost in the form of higher BLa and RPE on the cycle ergometer which therefore should be taken into account with loading and recovery prescription.

## Ethics Statement

This study was carried out in accordance with the recommendations of National Statement on Ethical Conduct in Human Research, University of Canberra, Human Research Ethics Committee (HREC 17-63). The protocol was approved by the University of Canberra, Human Research Ethics Committee. All subjects gave written informed consent in accordance with the Declaration of Helsinki.

## Author Contributions

JL was accountable for all aspects of the work. AR planned and designed the study, performed data collection, analysed the data and edited the manuscript. NV performed data collection, analysed the data and edited the manuscript. AM and MW edited the manuscript.

## Conflict of Interest Statement

The authors declare that the research was conducted in the absence of any commercial or financial relationships that could be construed as a potential conflict of interest.
